# The Role of Long Non-Coding RNAs in the Tumor Immune Microenvironment

**DOI:** 10.3389/fimmu.2022.851004

**Published:** 2022-02-09

**Authors:** Yingli Guo, Yajuan Xie, Yao Luo

**Affiliations:** ^1^State Key Laboratory of Biotherapy and Cancer Center, West China Hospital, Sichuan University, and Collaborative Innovation Center for Biotherapy, Chengdu, China; ^2^Department of Orthodontics, Hospital of Stomatology, Sun Yat-sen University, Guangzhou, China

**Keywords:** long non-coding RNAs, tumor immune microenvironment, cancer immunotherapy, biomarker, targeted therapy

## Abstract

Tumorigenesis is a complicated process caused by successive genetic and epigenetic alterations. The past decades demonstrated that the immune system affects tumorigenesis, tumor progression, and metastasis. Although increasing immunotherapies are revealed, only a tiny proportion of them are effective. Long non-coding RNAs (lncRNAs) are a class of single-stranded RNA molecules larger than 200 nucleotides and are essential in the molecular network of oncology and immunology. Increasing researches have focused on the connection between lncRNAs and cancer immunotherapy. However, the in-depth mechanisms are still elusive. In this review, we outline the latest studies on the functions of lncRNAs in the tumor immune microenvironment. *Via* participating in various biological processes such as neutrophil recruitment, macrophage polarization, NK cells cytotoxicity, and T cells functions, lncRNAs regulate tumorigenesis, tumor invasion, epithelial-mesenchymal transition (EMT), and angiogenesis. In addition, we reviewed the current understanding of the relevant strategies for targeting lncRNAs. LncRNAs-based therapeutics may represent promising approaches in serving as prognostic biomarkers or potential therapeutic targets in cancer, providing ideas for future research and clinical application on cancer diagnosis and therapies.

## 1 Introduction

The immune system and cancer progression are tightly connected. When immune cells recognize exogenous threats or endogenous mutations, they respond to changes in the microenvironment, from a static sentinel role to an active responder. For example, the cancer antigens are presented to T-cells *via* antigen-presenting cells (APCs). T-cells are activated after recognizing cancer cells and allow effector T-cells, other endogenous immune cells, and antibodies to eliminate cancer cells (1–3). However, the limited antigen recognition, pro-tumor phenotype differentiation, immune suppression, and impaired immune cell functions are all contributors to cancer development (4–6). Therefore, it is indispensable to improve the understanding of tumor immunology.

LncRNAs have revealed the diverse regulatory roles in immune responses and cancer progression. Different from mRNAs, the biogenesis of lncRNAs is associated with their specific subcellular localizations and functions. Depending on their localization and their specific interactions with DNA, RNA, and proteins, lncRNAs can modulate chromatin function, regulate the stability and translation of cytoplasmic mRNAs and interfere with signaling pathways. LncRNAs participated in immune activities such as antigen presentation, immune cells activation, and immune responses (7–10).

Cancer immunotherapy has aroused increasing interest. Unlike conventional systemic therapies that are directly cytotoxic to tumor cells, cancer immunotherapy depends on the immune system to set antitumor effects. Therefore, various factors that may affect host tumor immunity should be considered for proper design (11). LncRNAs are encouraged to be incorporated into cancer immunotherapy investigations, given their critical involvement in shaping immune responses. Targeting lncRNAs represents an attractive approach for potential biomarkers and therapeutical strategies, owning to its condition-specific expression pattern (12). A better understanding of lncRNAs-mediated approaches will provide new insights into the diagnosis and therapeutic strategies.

In this review, we summarized functions and regulating mechanisms of lncRNAs in in tumor immune microenvironment from various perspectives. In addition, we reviewed the current understanding of the relevant strategies for targeting lncRNAs. This review highlighted the essential roles of these lncRNAs-mediated approaches, which have great potential in immunotherapies to facilitate future cancer diagnosis and treatment.

## 2 Biogenesis and Functions of LncRNA

Non-coding RNA refers to a functional RNA molecule that can hardly be translated into protein. A large number of researches have shown that non-coding RNA plays an increasingly important role in the regulation of epigenetics. Common non-coding RNAs with regulatory effects include small interfering RNA, miRNA, piRNA, and lncRNA. LncRNA is a kind of non-coding RNA with a length greater than 200 nucleotides. The origin of lncRNA is still not clear, and the possible origins have been demonstrated: (a) Mutations of protein-coding genes; (b) Chromosomal rearrangement: lncRNA can be produced by recombination of separated gene sequences; (c) Duplications: The adjacent structural units in the lncRNA sequence are repeated, increasing the length of the transcript; (d) Transposon insertion: Inserting transposable elements containing transcription initiation sites into the genome to produce functional lncRNA sequences (13–16). According to the relative position of lncRNA coding sequence and protein-coding gene, it can be divided into several categories: sense lncRNA, antisense lncRNA, bidirectional lncRNA, intronic lncRNA and intergenic lncRNA (9, 17, 18).

Although lncRNA was initially thought to be a by-product of RNA polymerase II transcription, a kind of “noise” without biological function. Although lncRNA generally has no protein-coding ability actually, some of them can encode some short peptides. In recent years, lncRNA has become a research hotspot. LncRNA has a similar structure to mRNA. After splicing, it has a poly-A tail and promoter structure. There are dynamic expressions and different splicing methods during the differentiation process to form different lncRNA. Moreover, the conservation of lncRNA is low. The expression of lncRNA has tissue specificity and spatiotemporal characteristics. LncRNA expression varies from different tissues, different growth stages, and different locations (19–24). The features of lncRNAs have been studied and shown that lncRNAs play a vital role in many life activities such as dosage compensation effect, epigenetic regulation, cell cycle regulation, and cell differentiation (17, 25).

**Figure 1:**
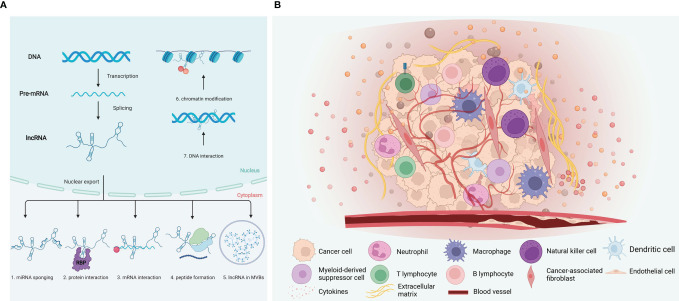
Mechanisms of lncRNA function and tumor immune microenvironment. **(A)** LncRNA regulate their targets *via* various mechanisms, including both the transcriptional and post transcriptional levels. (1) MicroRNA sponge; (2) interaction with protein; (3) interaction with mRNA; (4) peptide formation; (5) lncRNA in MVBs; (6) Chromatin modification; (7) DNA interaction. **(B)** Components of the tumor immune microenvironment. The tumor immune microenvironment is a complex ecosystem, consist of cancer cells, immune cells, and vascular network, etc. Induced by the cytokines, chemokines, and growth factors, tumor-infiltrating immune cells of both the myeloid and lymphoid lineages are recruited and activated. Created with BioRender.com.

LncRNAs participate in regulating various processes in the nucleus and cytoplasm, having a powerful regulatory effect in gene expression, and exerting cellular effects through various mechanisms. LncRNAs elicit functional outcomes through modular domains to interact with DNA, RNA, various regulatory proteins, and signaling (**Figure 1A**) (26, 27): (a) Transcription level. Local lncRNAs regulate the expression of adjacent protein-coding genes through transcription programs. The lncRNA transcript can regulate the act of transcription or splicing of the lncRNA can generate a chromatin state or steric impediment that influences the expression of nearby genes (10, 28). (b) Epigenetic modification level. Epigenetic modification includes histone and DNA methylation, histone acetylation and ubiquitin-like. Both cis-acting and trans-acting nuclear lncRNAs establish interactions with DNA to alter the chromatin environment or binding DNA in a sequence-specific manner (10). (c) Post-transcriptional level. LncRNAs can regulate post-transcriptional mRNA functions by complementary base pairing with target mRNA. The formation of RNA duplexes between complementary lncRNA and mRNA may conceal essential mRNA required for binding reaction factors, potentially affecting post-transcriptional gene expression, including mRNA precursor processing, transport, translation, and degradation (29). MicroRNA (miRNA) can directly bind to mRNAs by specific identification in a base-pairing manner, and thus inducing mRNA degradation at the post-transcriptional level by forming RNA-induced silencing complex (RISC) with related proteins such as Argonaute 2 (AGO2). LncRNAs act as competing endogenous RNAs (ceRNAs) to harbor the miRNA response elements (MREs) with complementary miRNA binding sites, sponging miRNA or keeping miRNAs away from mRNAs (30); (d) LncRNA–protein. LncRNAs interact with proteins, serving as molecular scaffolds, guides, or decoys to modulate protein function and interactions (31–34).

Based on these characteristics, lncRNAs can be used as a critical regulator in many aspects of biological activities, such as regulating cell proliferation, differentiation, and apoptosis. The regulatory roles of lncRNAs on immune cells and tumor cells have become a new direction for studying cancer immunology.

## 3 Tumor Immune Microenvironment

The tumor immune microenvironment (TIME) has received significant attention in recent years. TIME comprises tumor cells, immune cells, tumor-related fibroblasts, surround micro-vessels, various cytokines, and extracellular matrix (ECM) (**Figure 1B**) (6). TIME is a complex integrated system. If tumor cells are considered as seeds, then the microenvironment would act as soil. Tumor cells and their microenvironment interact and evolve together, affecting the generation and progression of tumors (35).

The tumor microenvironment presents the characteristics of hypoxia. Due to insufficient oxygen supply, tumor cells mainly undergo anaerobic glycolysis, leading to lactic acid accumulation (36). Meanwhile, ion-exchange proteins on the tumor cell membrane also continuously transport H+ outside the cells. These cellular responses cause the pH of the tumor microenvironment to decrease. In the hypoxic and acidic microenvironment, tumor tissues and peripheral tissue cells will undergo apoptosis, releasing cell debris and chemokines, leading to infiltration of inflammatory cells and secretion of inflammatory factors. In addition, the tumor itself can also trigger an immune response, causing inflammatory cells to accumulate in this area, triggering the inflammatory response (37, 38).

Tumor cells and tumor microenvironment complement each other. The tumor immune microenvironment is closely related to the efficacy of immunotherapy. Tumors can affect their microenvironment by releasing signaling molecules, promoting tumor angiogenesis and immune tolerance. Immune cells and other components in the microenvironment can affect the growth and development of cancer cells (35). For example, the infiltrated immune cells participate in the immunosuppressive tumor microenvironment formation to facilitate immune escape and malignant development. Among them, regulatory T cells (Tregs) play a significant role. Macrophages have different subtypes in the tumor microenvironment. Studies have shown that M1-type macrophages mainly inhibit tumors, promoting inflammation and immune activity. In contrast, M2-type macrophages play a role in tissue repair, immune escape, and promote tumor progression (39, 40). In addition to immune cells, many non-immune components constitute the tumor microenvironment, such as fibroblasts, vascular endothelial cells, and other stromal cells. Cancer-associated fibroblasts (CAFs) can release stromal cell-derived factors and pro-angiogenesis factors to promote tumor cell growth and metastasis. Vascular endothelial cells mainly mediate tumor angiogenesis (41, 42). By learning tumor immune microenvironment, clinical diagnosis and treatment can be better guided, and precision medicine can be realized.

## 4 Tumor Immune Escape

The host’s immune system has the function of immune surveillance to recognize and specifically eliminate these “non-self” cells through the immune mechanism to resist tumorigenesis and tumor development (43). However, in some cases, malignant cells can evade the body’s immune surveillance, escape the recognition and attack of the body’s immune system through various mechanisms (43, 44). The in-depth study of tumor immune escape mechanisms provides new ideas for the exploration of tumor immunotherapy (5, 44). Mechanisms resulting in immune escape include the selection of tumor variants resistant to immune effectors and the tumor immune-suppressive environment (45).

Immune escape can be achieved through intrinsic tumor properties or the tumor microenvironment, that include (1) Low expression of tumor-associated antigens. Tumor-specific CD8+ T cells are activated by recognizing tumor antigens, depending on the specific recognition and binding of TCR to MHC-I-peptide complexes. Decreased expression of tumor-associated antigens affects the recognition of MHC molecule antigen peptide complexes by TCR (46, 47). (2) Low expression of MHC molecules. The lack of presentation of MHC-I molecules is often one of the main reasons for tumor immune escape (48). (3) Tumor cells lack costimulatory molecules. Even though tumor cells can directly present tumor antigens to T cells through the MHC molecules, they cannot activate T cells due to the lack of costimulatory signals, resulting in T cell immune response and even tolerance induction (49). (4) Tumor apoptosis is suppressed. The Fas/FasL system is of great significance in immune escape. Fas belongs to the TNF receptor family, and its ligand FasL can mediate cell apoptosis. On the one hand, the tumor cells themselves only express a little or no Fas to be protected from attack by themselves or immune cells. On the other hand, the tumor cells actively express FasL to kill the infiltrating Fas-positive effector cells (50, 51).

Tumor-related immunosuppressive factors in the tumor microenvironment are the contributors to immune escape. Immune checkpoint molecules are inhibitory regulatory molecules expressed on immune cells, suppressing the effective anti-tumor immune response. Under physiological conditions, immune checkpoint molecules regulate the immune system, dampening the immune response after mitigating an infection or other threats (52). However, these immune checkpoint interactions may also be engaged in the development of cancer (53, 54). The immune checkpoint molecules related to tumors mainly include PD1, CTLA4, Tim3, LAG3, etc. Currently, PD1 and CTLA4 are mainly studied. Programmed death molecule 1 and its ligand (PD-1/PD-L1) are a pair of negative immunostimulatory molecules (55). Under normal circumstances, after PD-L1 is combined with PD-1 on the surface of lymphocytes, it can inhibit lymphocyte function and induce apoptosis of activated lymphocytes, thereby exerting autoimmune tolerance. However, PD-L1 is also expressed on the surface of many tumor cells. PD-L1 expressed by tumor cells can bind to PD-1 on the surface of corresponding lymphocytes, inhibit the function of lymphocytes and the release of cytokines, lead to lymphocyte apoptosis and immune escape of tumor cells (56). In recent years, immunotherapy has been considered a promising treatment strategy, which utilizes the host’s immune system to combat malignant cells by inhibiting the immune checkpoint pathway. Therefore, blocking the PD-1/PD-L1 pathway can enhance the activity of lymphocytes to achieve the effect of tumor immunotherapy (57). Cytotoxic T lymphocyte-associated antigen-4 (CTLA-4), also known as CD152, is a leukocyte differentiation antigen, a transmembrane receptor on T cells, and it shares B7 with CD28 Molecular ligands. The binding of CTLA-4 and B7 molecules induces T cell anergy, which participates in the negative regulation of immune response. Immune checkpoint inhibitors are some monoclonal antibodies developed for corresponding immune checkpoints. Their primary function is to block the interaction between tumor cells expressing immune checkpoints and immune cells, thereby blocking the effect of tumor cells on immune cells (58, 59).

In the tumor microenvironment, there are many tumor-related immunosuppressive cells, including regulatory T cells (Treg), tumor-associated macrophages (TAM), and myeloid-derived suppressive cells (MDSC), etc. The chemokines in the tumor microenvironment recruit the Tregs in the thymus, bone marrow, lymph nodes, and the periphery to the tumor through the Treg receptor CCR4, thereby suppressing immunity. Tumor-associated macrophages (TAM) in tumor tissues actively inhibit the immune response and plays a role in tumor development. They also facilitate the expression of epidermal growth factor (EGF), platelet-derived growth factor (PDGF), TGF-B, hepatocyte growth factor (HGF), and matrix metalloproteinases (MMPs), directly promoting tumor progression. Myeloid-derived suppressor cells (MDSC) can express a variety of pro-angiogenic factors, directly promote tumor angiogenesis. Also, MDSC can inhibit T cell-mediated adaptive anti-tumor immunity through the high expression of ARG1, iNOS, and ROS (60, 61).

## 5 LncRNAs Regulate Tumor Immune Microenvironment

### 5.1 LncRNAs and Innate Immune Cells in TIME

**Figure 2:**
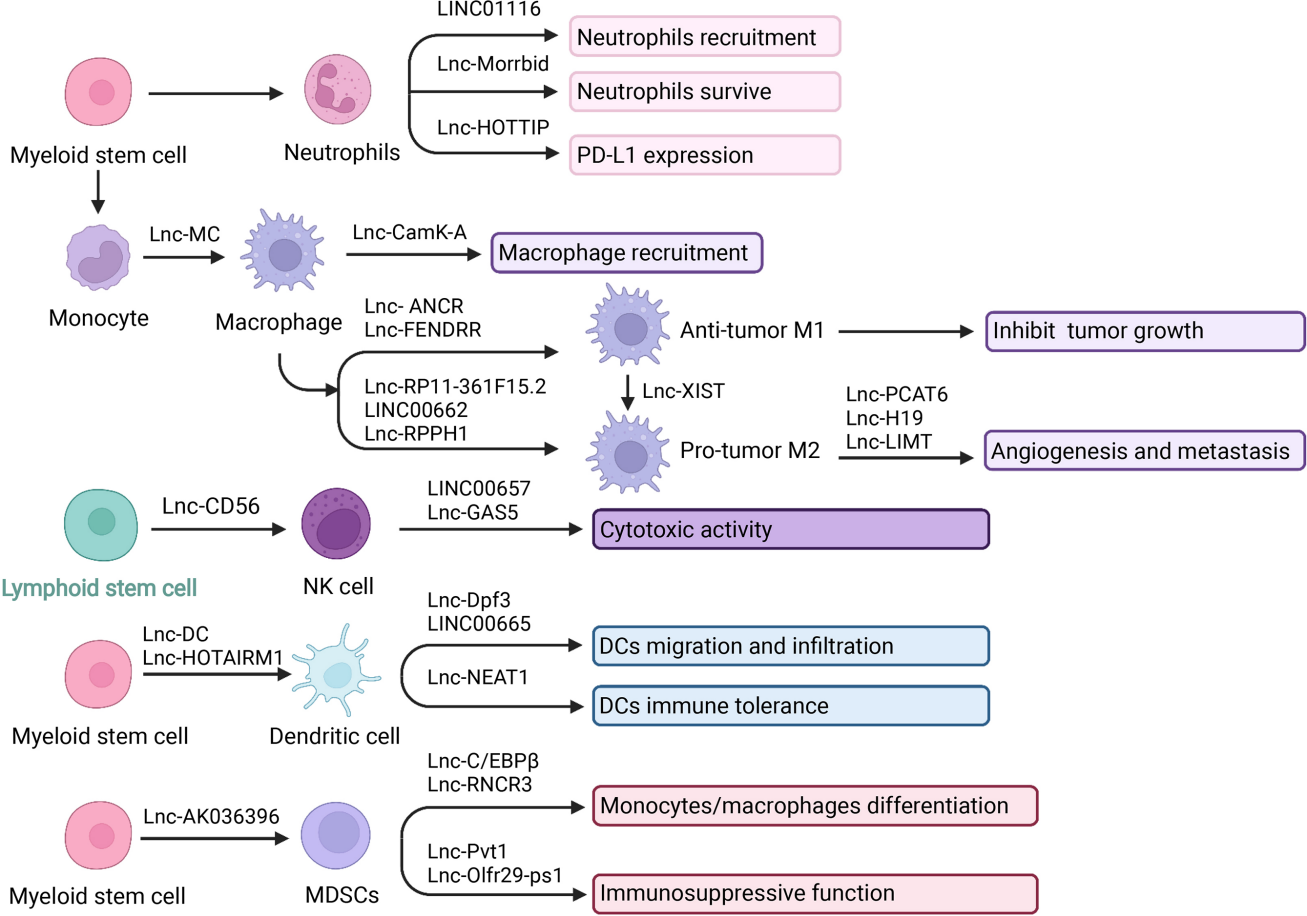
LncRNA and innate immune cells in TIME. LncRNA is central player in cancer biology and they play a pivotal role in mediating the network communication between tumor cells and their microenvironment. Innate immune effectors including neutrophils, macrophages, NK cells, DCs, and MDSCs. LncRNA can interact with tumor-infiltrating innate immune cells to modulate their recruitment, development and function in tumor microenvironment. Created with BioRender.com.

The innate immune system is the first defense line, relying on a surveillance system of neutrophils, macrophages, natural killers, and dendritic cells, which recognize and act on pathogens non-specifically. In addition, they also activate the adaptive immune system through antigen presentation (62). Recently, lncRNAs have been documented to be associated with innate immune cell development, differentiation, and immune responses (**Figure 2**). LncRNAs have emerged to act functionally through modular domains, regulating gene expression and modulating pathogen response pathways *via* interactions with chromatin, RNA, and proteins (18). Taken together, considering the critical roles of lncRNAs, it makes sense to explore the lncRNAs-mediated regulation in the TIME.

#### 5.1.1 LncRNAs and Neutrophils

Neutrophils derived from myeloid progenitor cells participate in natural immunity and regulate adaptive immunity (63, 64). Affected by the tumor microenvironment, neutrophils have different polarization states. Different polarization states have different effects on tumors, which can promote or inhibit tumor growth. Anti-tumor N1 type and tumor-promoting N2 type are the two main polarization types of neutrophils. The polarization of N1 neutrophils is usually induced by IFNs, IL-1β, IL-8, TNF-α. While N2 neutrophils are induced by TGF-β, IL-8, IL-6, and IL-17 (65, 66). The anti-tumor mechanisms of neutrophils include: (a) N1 neutrophils directly kill tumor cells by releasing reactive oxygen species (ROS) and reactive nitrogen (RNS); (b) As antigen-presenting cells, neutrophils can directly stimulate T cell activation, and simultaneously release a variety of chemokines such as TNF-α, and cathepsin G to activate DCs, macrophages, NK cells, and T cells. Neutrophils have a synergistic effect with CD8+ T cells in the anti-tumor process, while Tregs limit the infiltration of neutrophils; (c) After the adaptive immune response is initiated, N1 neutrophils kill tumor cells through antibody-dependent cell-mediated cytotoxicity ADCC (67, 68). The pro-tumor mechanisms include: (a) N2 neutrophils secrete cytokines such as VEGF and TNF to promote tumor angiogenesis; (b) Neutrophils secrete matrix metalloprotein 9 (MMP-9) to degrade type IV collagen of the basement membrane, promoting the infiltration of tumor cells. In addition, MMPs also participate in the reconstruction of tumor extracellular matrix and promote tumor progression; (c) Granule colony stimulating factor (G-CSF) and TGFβ can induce neutrophils to express arginase 1 (ARG1), ROS and nitric oxide (NO), to inhibit T cell activation; (d) Recruit anti-inflammatory M2 macrophages (67, 69, 70).

It has been demonstrated that lncRNAs participated in neutrophil recruitment, lifespan, and function. Knockdown of LINC01116 affected the secretion of IL-1β, thereby promoting the recruitment of tumor-associated neutrophils (TAN). The accumulation of TAN produced a large number of cytokines to promote tumor proliferation (71). Moreover, neutrophils are short-lived immune cells with a half-life of 7-10 hours in human circulation. Regulation of neutrophil lifespan is critical in the tumor immune microenvironment. LncRNAs have also been revealed to regulate the neutrophils’ lifespan (65). LncRNA Morrbid was evidenced to regulate the survival status of neutrophils. Morrbid regulated the transcription of the neighboring pro-apoptotic gene Bcl2l11 by promoting the enrichment of the PRC2 complex at the Bcl2l11 promoter to maintain a poised state. Thus, regulating Morrbid allowed rapid control of apoptosis in response to extracellular pro-survival signals, representing a potential therapeutic target (72). Besides, regulatory role was also achieved *via* PD1/PD-L1 pathway. LncRNA HOXA transcript at the distal tip (HOTTIP) enhanced IL-6 expression to potentiate immune escape of ovarian cancer cells by upregulating the expression of PD-L1 in neutrophils. HOTTIP was noted to promote the expression of IL-6 by binding to c-jun, which resulted in a promoted PD-L1 expression in neutrophils and immune escape. Meanwhile, T cell proliferation and efficiency of tumor immunotherapy were inhibited (73). The infiltration of neutrophils in patients with gastric cancer was significantly increased, showing an activated CD54+ phenotype and expressing high levels of immunosuppressive molecule PD-L1. A significant correlation was found between the levels of PD-L1 and CD54 on tumor-infiltrating neutrophils. Tumor-derived GM-CSF activates neutrophils and induces PD-L1 expression in neutrophils through the Janus kinase (JAK) and signal transducer and activator of the transcription 3 (STAT3) signaling pathway. The binding of PD-1 to its ligand PD-L1 is essential for the physiological regulation of the immune system. Activated PD-L1+ neutrophils effectively inhibit normal T cell immunity *in vitro* and promote tumor growth and progression (74, 75).

#### 5.1.2 LncRNAs and Macrophages

Macrophages residing in tissues can differentiate from circulating monocytes or from the fetal liver and yolk sac during embryonic development. Macrophages are the critical effector cells of innate immunity, having powerful phagocytosis. In addition, they can also release cytokines and chemokines to recruit antigen-presenting cells or T cells to initiate an adaptive immune response (76–79). Depending on microenvironmental stimuli, macrophages are able to have different phenotypes. M1 phenotype and M2 phenotype are two principal polarizations. M1-type macrophages can kill tumor cells and resist pathogen invasion, and M2-type macrophages mainly promote tumor growth, invasion, and metastasis. Macrophages in tumor tissues mostly have the M2 phenotype and function. Therefore, the specific M2 morphological macrophages that exert immunosuppressive and tumor-promoting effects are narrowly defined as tumor-associated macrophages (TAM) (80, 81). LPS and IFN-γ signals are M1 stimulation to induce the activation of NF-κB (p65 and p50), AP-1, IRF3, and STAT1 through the TLR4, IFN-α, IFN-β, and IFN-γ receptor pathways, triggering M1 polarization (82–84). IL-4 and IL-13 are M2 stimulation to activate STAT6 through IL-4Rα signal; IL-10 activates STAT3 through IL-10R signals to trigger M2 polarization (84). M1 macrophages have pro-inflammatory properties and inhibit tumor growth: (a) Direct killing effect; (b) Antibody-dependent cell-mediated cytotoxicity (ADCC): Macrophages expressing IgGFc receptors can kill the target cells by binding to the Fc segment of IgG antibodies on the surface of tumor cells; (c) Induce specific immune response: Macrophages present antigens to promote Th1 immune response (85, 86). M2 macrophages have anti-inflammatory properties and promote tumor growth: (a) Promote tumor cell proliferation; (b) Promote angiogenesis; (c) Participate in tumor cell infiltration, and metastasis; (d) Suppress immune function; (e) Chemotherapy resistance (87–89). Understanding the regulatory role and function of lncRNAs in macrophages activities and cancer immunity will help to explore potential diagnosis and treatment methods.

Recently, there have been increasing studies about lncRNAs in macrophage differentiation, recruitment, polarization, and functions. Activin A receptor type 1B (ACVR1B) plays an essential role in the monocyte and macrophage differentiation process. Lnc-MC was reported to participate in macrophage differentiation *via* regulating ACVR1B expression. Mechanistically, lnc-MC as a competing endogenous RNA can interact with microRNA 199a-5p. Then the repression of ACVR1B expression was alleviated. Consequently, the transforming growth factor β (TGF-β) signal pathway was activated, and monocyte/macrophage differentiation was facilitated (90, 91). In addition, there were reports about lncRNAs in macrophage recruitment. Calcium (Ca2+) flux and Ca2+-dependent signaling play key roles in tumor growth and progression. A study revealed that lncRNA calcium-dependent kinase activation (lncRNA CamK-A) was highly expressed in cancer and participated in macrophage recruitment and microenvironment regulation by activating Ca2+-triggered signal transduction. In terms of mechanism, CamK-A activated Ca2+/calmodulin-dependent kinase PNCK, which in turn phosphorylated IκBα and activated calcium-dependent nuclear factor κB (NF-κB). The expression of CamK-A was coordinated with the activation of the CaMK-NF-κB axis. This pathway led to the recruitment of macrophages and angiogenesis. Moreover, CamK-A can affect cancer development. Clinically, the high expression of CamK-A was associated with poor prognosis, indicating that it can be used as a potential biomarker and therapeutic target (92).

Considering M1-type macrophages inhibit tumor growth, the roles of lncRNAs in M1 polarization have been appreciated. FOX protein is a transcription factor with a highly conserved sequence, which can facilitate macrophages to release inflammatory factors, cause M1 polarization, and achieve the anti-tumor effect. In one study, lncRNA ANCR was reported to target FoxO1 and inhibit the expression of FoxO1 by promoting ubiquitination and degradation of FoxO1. The overexpression of lncRNA ANCR can inhibit the polarization of M1 type, thereby further promoting the invasion and migration of cancer cells (93). Furthermore, in another study, lncRNA fetal-lethal non-coding developmental regulatory RNA (FENDRR) was associated with M1 macrophages. Overexpression of FENDRR facilitated M1 macrophage polarization, while knockdown of FENDRR down-regulated M1 type (94).

TAM has an M2-like effect and can promote tumor development and angiogenesis. Studies have revealed the connection between lncRNAs and M2 polarization. Previous studies have reported that lncRNAs can act as ceRNA to protect the target mRNAs expression, keep miRNAs away from mRNAs, or *via* extracellular vesicles (EVs) pathways to regulate M2-like polarization (18, 95). For example, cytoplasmic polyadenylation element binding protein 4 (CPEB4) has been identified as related to M2-Like polarization. LncRNA RP11-361F15.2 acted as ceRNA to sponge miR-30c-5p, thereby binding and activating CPEB4, increasing osteosarcoma progression and metastasis (96). Also, in another study, LINC00662 competitively bound to miR-15a, miR-16, and miR-107 to upregulated WNT3A expression thus activated Wnt/beta-catenin signaling in macrophages and further promoted M2 polarization and tumor progression (97). Exosomes are small membrane-bound vesicular particles participating in intercellular communication and regulation (98). LncRNAs can be packaged into the vesicles. Studies have found that the exosome derived from macrophages can influence the development of disease by carrying lncRNAs. LncRNA RPPH1 overexpression was associated with promoting colorectal cancer (CRC) metastasis. CRC cell-derived exosomes can transport RPPH1 to macrophages, thereby mediating M2 polarization (99). Furthermore, M2 macrophages is also involved in the angiogenesis, aggressiveness, and metastasis of cancer. For example, the upregulation of prostate cancer-related transcript 6 (PCAT6) accelerated angiogenesis in triple-negative breast cancer (TNBC). PCAT6 upregulated the expression of VEGFR2 through the ceRNA model and then promoted angiogenesis through the VEGFR/AKT/mTOR signaling pathway. In addition, PCAT6 combined with the deubiquitinating enzyme USP14 to induce deubiquitination of VEGFR2, which also promoted angiogenesis (100). The long non-coding RNA H19 induced by macrophages activated the miR-193b/MAPK1 pathway, thereby promoting the aggressiveness of hepatocellular carcinoma (101). Besides the above, M2 macrophages can secrete epidermal growth factor (EGF) to suppress lncRNA inhibiting metastasis (LIMT) expression through the EGFR-ERK axis, thus promoting ovarian cancer metastasis (102).

M1-M2 conversion is another hot spot of concern. LncRNA X inactivation specific transcript (XIST) was reported associated with M1-M2 polarization. The absence of XIST increased the secretion of exosomes miRNA-503, which can induce M1-M2 polarization of microglia, thereby upregulating immunosuppressive cytokines, inhibiting T cell proliferation, then accelerating primary tumor growth and metastasis (103). In another study, the knockdown of XIST induced the conversion of M1 to M2 by inhibiting the expression of enhancer binding protein (C/EBP) α and Kruppel-like factor 6 (KLF6), thereby promoting the proliferation and migration of tumor cells (104).

All these studies have shown a close connection between lncRNAs and the differentiation, recruitment, and polarization of macrophages. At the same time, their interaction may affect the occurrence, development, invasion, metastasis, and vascularization of tumors. A deep understanding of these relationships is significant for exploring the biomarkers, diagnosis, and treatment of cancer.

#### 5.1.3 LncRNAs and Natural Killer Cells

NK cells are the body’s first line of defense against cancer cells and virus infections. They can directly kill tumor cells non-specifically. This natural killing activity does not require antigen sensitization nor antibody participation, and there is no MHC restriction. In addition to having a powerful killing function, it also has immune regulation function, interacting with other immune cells in the body and regulating their immune state and immune function. Clinical studies have found that NK cell adoptive immunotherapy has good application prospects for malignant tumors (105–108). At present, it is believed that NK cells mainly exert their killing effect through the following ways. Firstly, NK cells directly release cytotoxic particles such as perforin and granzyme through exocytosis and activate the caspase pathway to induce target cell apoptosis (109). Cells express a series of stimulating and inhibiting receptors, and subsequent reactions occur after being combined with specific ligands (such as PD1-PDL1, NKG2-HLA, CD28H-B7H7, and DNAM1-CD155). The expression levels of tumor ligands and NK cell receptors determine whether NK cells will kill tumor cells (110). Also, the cytokine-mediated killing effect is essential. NK cells can synthesize and secrete various cytokines, such as IFNγ, TNFα, IL1, IL5, IL8, IL10, and GCSF (109). For example, secreted interferon-γ (IFNγ) can increase the expression of human leukocyte antigen (HLA) class I in tumor cells, thereby enhancing the presentation of tumor antigens to T cells and exerting the killing effect of T cells (105). The NK cells infiltration in the TME is related to a good prognosis, showing the potential in malignancies treatment (111). However, an accumulation of tumor-derived inhibitory molecules such as adenosine and lactate may limit NK cell functionality in the immunosuppressive TME (112).

Nowadays, increasing studies have focused on the role of lncRNAs in NK cells development, differentiation stages, and the relevance in regulating cancer immunity (113). Lnc-CD56 was reported to regulate CD56 and affect NK cells’ maturity positively. Lnc-CD56 can interact with the transcription factors such as TBX21, IRF2, IKZF2, ELF4, and EOMES of the NK cells. Then the CD56 expression and NK cell development were promoted. While knockdown lnc-CD56 down-regulated CD56 transcription and reduced mature CD56+ NK cells (114–116).

In addition, the cytotoxic activity of NK cells can also be regulated by long non-coding RNA. Death receptors (DRs) DR5 is an essential inhibitor of immune tolerance and able to enhance the cytotoxicity of NK cells. Increasing expression of DR5 can be achieved by overexpression of RUNX3 (117). Furthermore, the up-regulation of RUNX3 can be achieved by LINC00657 down-regulating miR-20a-5p. Therefore, LINC00657 can enhance the cytotoxicity of NK cells to cervical cancer cells through miR-20a-5p/RUNX3/DR5 axis to inhibit tumor proliferation and metastasis (118). Moreover, lncRNAs can regulate cytokines secretion, which also contributes to the killing effect of NK cells (119). IFN-γ secretion was associated with the increased cytotoxicity of NK cells. In a study, overexpression of lncGAS5 can increase IFN-γ secretion through the miR-544/RUNX3 axis. LncRNA GAS5 up-regulated the expression of RUNX3 by negatively regulating miR-544, thereby promoting the secretion of IFN-γ and enhancing the killing effect of NK (120). Similar in another study, GAS5 promoted the secretion of IFN-γ and TNF-α by regulating miR-18a, thereby enhancing the killing effect of NK cells on gastric cancer (GC) (121). NK cells are essential in enhancing immune function and inhibiting immune escape. Taking full advantage of the immune function of NK cells to kill tumors is a hot research area. Considering the critical impact of lncRNAs on the function of NK cells, strategies based on lncRNAs may guide anti-tumor therapies.

#### 5.1.4 LncRNAs and Dendritic Cells

Dendritic cells are the body’s most potent antigen-presenting cells, derived from Hematopoietic Stem Cells (HSC). Dendritic cells are responsible for recognizing danger-associated molecular patterns (DAMPs) or pathogen-associated molecular patterns (PAMPs). They can efficiently ingest, process, and present antigens. DCs are the central link of initiating and regulating immune response (122, 123). The main task of dendritic cells is to carry tumor antigens and present them to T cells to activate the anti-tumor function (124). In addition, dendritic cells can also stimulate the proliferation and maturation of B lymphocytes, stimulate the activation of Th cells and NK cells, and activate the immune functions in various ways. After dendritic cells take up tumor antigens, they are delivered to CTLs (cytotoxic T lymphocytes) *via* MHC-I or MHC-II pathways. At the same time, a set of costimulatory signals are provided, such as B7/CD28, IFA-3/CD2, ICAM-1/IFA-1, which fully activate CTL and produce an anti-tumor immune response (125–127). Moreover, DC can induce the differentiation and maturation of NK cells. And also, NK cells can induce the maturation of immature DCs. Activated NK cells are the first line of defense for human immunity (128). DC cells act as the commander-in-chief during the entire immune process. DCs mobilize other immune cells in the body to perform tumor-killing functions, which is conducive to tumor clearance. LncRNAs have been reported to participate in the antigen presentation and pathogen-response pathways, playing a role in DCs differentiation, migration, and function.

LncRNAs have been reported to be related to the differentiation of DCs. Knockout of lnc-DC interfered with DC differentiation and affected the activation and function of T cells. Lnc-DC regulated this series of responses by activating the transcription factor STAT3. Lnc-DC directly interacted with STAT3 to promote phosphorylation of STAT3 on Tyrosine 705, by preventing STAT3 from binding to SHP1 and dephosphorylation of SHP1 (129). Moreover, as another research announced, the overexpression of lnc-DCs led to the over-maturation of dendritic cells and increased activation of Th1 cells. What’s more, lncRNAs regulated monocyte/dendritic cell differentiation (130). LncRNA HOTAIRM1 and miR-3960 affected the expression of myeloid differentiation-related HOXA genes. It formed a ceRNA network, which acted as a negative regulator of DC differentiation, enabling cells to maintain the monocyte phenotype without transforming into DCs (131).

The glycolytic metabolism and migration ability of DCs were also regulated by lncRNAs. The lnc-Dpf3 directly interacted with HIF-1α and inhibited the transcription of the HIF-1α-dependent glycolytic gene Ldha. Thereby DC glycolytic metabolism and migration ability were suppressed (132). In another study, LINC00665 overexpression was reported to correlate with a low infiltration level of DCs (133).

Moreover, previous results showed that lncRNAs could regulate DCs immune tolerance. Tolerogenic dendritic cells (tol-DCs) played an indispensable role in immune tolerance. LncRNA NEAT1 regulated the tolerogenic phenotype expression of DCs *via* the NEAT1/miR-3076-3p axis. Mechanistically, as a ceRNA, NEAT1 sponged miR-3076-3p to modulate the inflammasome NACHT, LRR, and PYD domains-containing protein 3 (NLRP3). NLRP3 was a key player in inflammation and associated with the activation of tol-DCs (134). These works have identified the roles of lncRNAs in regulating antigen presentation and DCs. Broadening the mechanisms of lncRNAs action and functions may provide new targets in novel strategies.

#### 5.1.5 LncRNAs and Myeloid-Derived Suppressor Cells

Myeloid-derived suppressor cells (MDSCs) are a type of heterogeneous cell derived from bone marrow precursor cells and immature myeloid cells (IMCs). Accumulation of MDSCs can promote tumorigenesis and tumor progression. They mediate tumor immune escape and promote tumor growth by inhibiting the function of effector T cells. Under normal circumstances, MDSCs are the precursors of DCs, macrophages, and granulocytes, quickly differentiating and entering the corresponding organs and tissues to exert normal immunity function. In tumor patients, immature precursor cells proliferate after MDSCs are stimulated by tumor-derived factors (TDFs). Then they inhibit the tumor-immune system and further promote the tumor development (135, 136). The main functional characteristic of MDSCs cells is their potent ability to suppress various types of immune responses (61). MDSCs have different differentiation potential and immunosuppressive abilities. MDSCs are mainly divided into two subgroups: monocytic-MDSCs (M-MDSCs) and granulocytic-MDSCs (G-MDSCs). M-MDSCs express ARG1 and iNOS to inhibit T cell effects by producing high nitric oxide. G-MDSCs mainly suppress the immune response in an antigen-specific manner and generate ROS (137, 138). Factors secreted by tumor cells and inflammatory cytokines produced by tumor stroma are the main signals leading to the accumulation of MDSCs. For example, stem cell factor (SCF), granulocyte-macrophage colony stimulating factor (GM-CSF), granulocyte colony stimulating factor (G-CSF), etc., stimulate the production and proliferation of MDSCs through the JAK-STAT signaling pathway. Transcription factors such as STAT3, STAT5, and NOTCH are involved in this process. Moreover, IFN-γ, IL-4, IL-6, IL-1β, and CXCL1 mainly induce the inhibitory activity of MDSCs through NF-κB, STAT1, and STAT6 (139, 140). The roles of lncRNAs in the maturation, differentiation and function of MDSC have been studied.

LncRNAs can regulate the development of MDSC. Ficolin B (Fcnb) expression can be evaluated as a surrogate for the development of polymorphonuclear myeloid-derived suppressor cells (PMN-MDSC) and is a predicted target gene of lncRNA F730016J06Rik (AK036396). Knockout of lncRNA AK036396 reduced the stability of Fcnb protein, which depended on the ubiquitin-proteasome system. LncRNA AK036396 inhibited the maturation of PMN-MDSCs and accelerated their immunosuppression by enhancing the stability of Fcnb protein (141).

LncRNAs can regulate the differentiation of MDSCs. Interleukin 4 induced gene 1 (IL4i1) played a vital role in the differentiation of monocytes/macrophages. Lnc-C/EBPβ interacted with C/EBPβ LIP and WDR5 to down-regulate IL4il, thereby affecting the differentiation of MDSCs (140). LncRNA RNCR3 acted as a competitive endogenous RNA to promote the differentiation of MDSCs and suppressive function through the RNCR3/miR-185-5p/Chop pathway (142).

In addition, lncRNAs also regulated the immunosuppressive ability of MDSCs. Studies have shown that knockdown of lncRNA Pvt1 reduced the levels of Arg1 and ROS in G-MDSC and partially restored the anti-tumor T cell response. LncRNA Pvt1 may be a potential therapeutic target for regulating the inhibitory function of G-MDSCs (143). LncRNA Olfr29-ps1 promoted the immunosuppressive function and differentiation of Mo-MDSCs by sponging miR-214-3p after modification with N6-methyladenosine (m6A). Moreover, lncRNA Olfr29-ps1 can be upregulated by IL6, which can be a potential anti-tumor target (144).

In short, MDSCs are critical immunosuppressive cells in the tumor immune microenvironment. MDSCs suppress the body’s immune cells through various channels, weaken the body’s killing effect on tumor cells, and contribute to the occurrence and development of tumors. Therefore, in-depth research and corresponding clinical practice of the interactions between lncRNAs and MDSCs still require more remarkable progress in the prevention of tumor immune escape.

### 5.2 LncRNAs and Adoptive Immune Cells in TIME

#### 5.2.1 LncRNAs and T Cells

**Figure 3:**
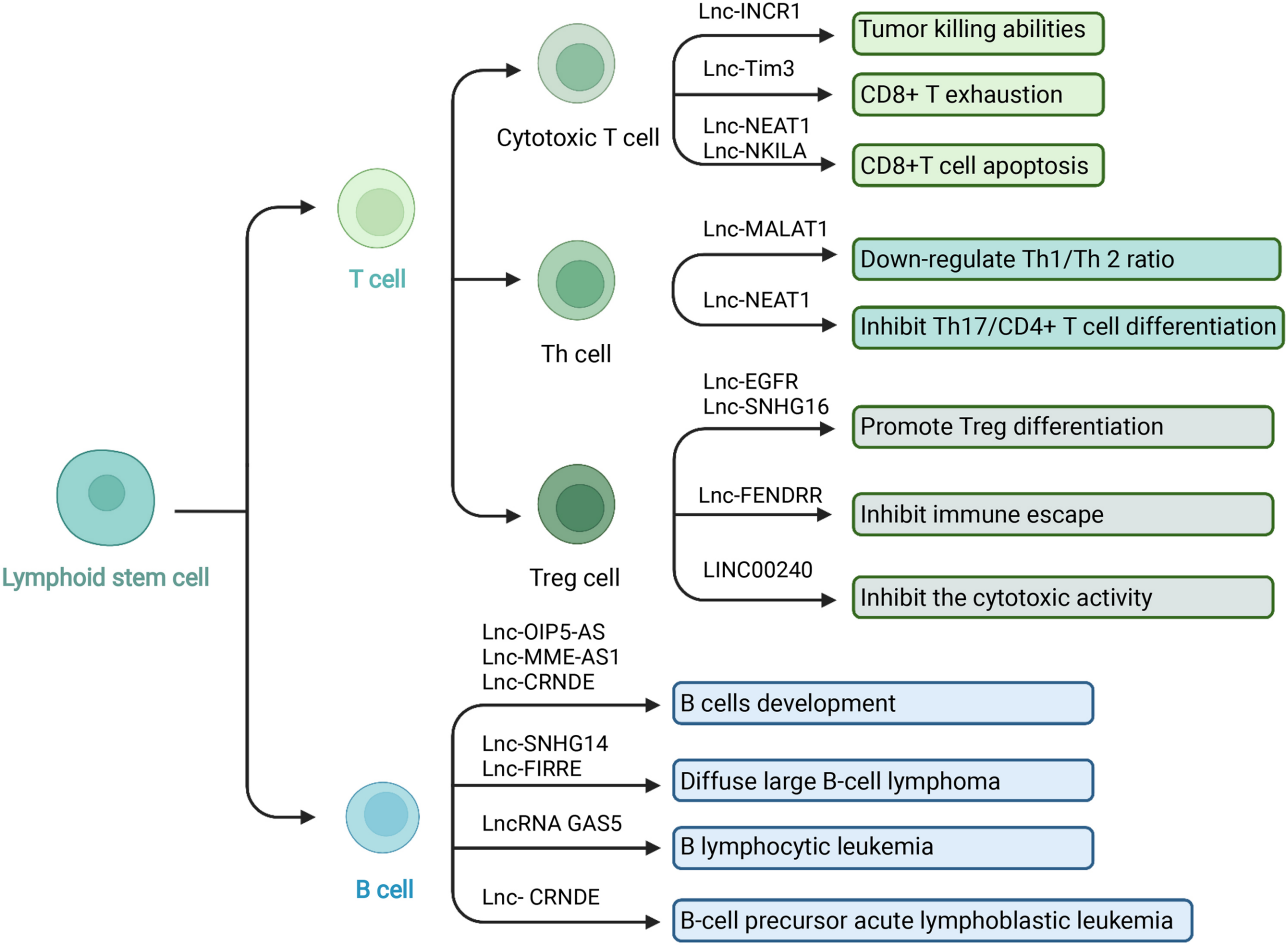
LncRNA and adaptive immune cells in TIME. In the TIME, lncRNA can regulate adaptive immune cells to interact with tumor cells. T cells and B cells are the main adaptive immune cells. T cells play different immunomodulatory effects depending the various subgroups. Cytotoxic T cells have the tumor killing abilities and Treg cells play an immunosuppressive role. LncRNA has been revealed to participate in adaptive immune cells’ development, activation, differentiation, function, and cancer immunology. Created with BioRender.com.

T lymphocytes are the main components of lymphocytes involved in adaptive immunity (**Figure 3**) (145). T cells have various subgroups and play different immunomodulatory effects. Cytotoxic T cells (CTLs) secrete perforin and granzyme to kill infected and mutant cells (146). T-helper (Th) cells have many classifications and various functions. For example, Th1 cells secrete IFN-γ, IL-2, TNF-α, and LN-α, which mediate the activation of macrophages and kill intracellular pathogens. Th1 cells play a crucial role in the anti-tumor immune response, while Th2 cells play an anti-Th1 cells role and promote tumor cell proliferation (147–149). Regulatory T cells (Treg) are an immunosuppressive subgroup of CD4+ T cells, which impair the immune monitoring against cancer and lead to tumor progression (39). Central memory T cells (Tcm) and effector memory T cells (Tem) are two main subtypes of memory T cells (Tm). Tcm can proliferate and differentiate into effector T cells when stimulated by antigens; the Tem can produce cytokines when stimulated by antigens (150). Activated natural killer T cell (NKT) is a T cell subgroup with T cell receptor (TCR) and NK cell receptor on the cell surface. They can quickly respond to antigen stimulation, produce multiple cytokines and chemokines, and have cytotoxic activity (151). LncRNAs have been revealed to participate in T cells’ development, activation, differentiation, function, and cancer immunology.

Cytotoxic T cells can specifically kill tumor cells and play an essential role in anti-tumor immunity. However, the tumor microenvironment can induce CD8+ T cells to increase the expression of the inhibitory receptor PD-1. After binding to the ligand PD-L1 on the surface of tumor cells, it significantly inhibits the ability of CD8+ CTL to eliminate tumor cells. Numerous basic investigations and clinical experiments have proved that anti-PD-1 or anti-PDL-1 methods can significantly enhance the body’s efficiency in removing tumors (53, 152, 153). For example, the expression of PD-L1 was upregulated by tumor IFN signals so that the immune surveillance function mediated by T cells was suppressed. Silencing long non-coding RNA IFN-stimulated non-coding RNA 1 (INCR1) controlled IFNγ by reducing the expression of PD-L1, JAK2, and several other IFNγ-stimulating genes. INCR1 knockdown made tumor cells more sensitive to cytotoxic T cell-mediated killing (154). TIM-3 (T cell immunoglobulin domain and mucin domain-3) is a type of T cell surface inhibitory molecule that can cause T cell death during cancer infection (155). Lnc-Tim3 has shown a regulatory role in CD8 T exhaustion. Mechanistically, lnc-Tim3 specifically interacted with Tim3 to suppress Tim-3–Bat3 signaling and downstream signaling pathway NFAT1 and AP-1 in CD8 T cells, leading to CD8+ T exhaustion (156, 157). In another study, the regulatory role of lncRNA NEAT1 in T cell function was also reported. *Via* the miR-155/Tim-3 pathway, repression of NEAT1 down-regulated the CD8+T cell apoptosis and enhanced the anti-tumor effects of T cells (158). Activation-induced cell death (AICD) is another major type of programmed death of T lymphocytes. After normal T lymphocytes are stimulated by invading antigens, T lymphocytes are activated and induce a series of immune responses. In order to prevent excessive immune response or prevent this immune response from developing indefinitely, AICD is used to control the lifespan of activated T cells. AICD can be used by cancer to avoid immune destruction. The association between lncRNA and AICD of T lymphocytes has also been reported. CTLs and TH1 were sensitive to AICD in breast and lung cancer. LncRNA NKILA regulated T cell sensitivity to AICD by inhibiting NF-κB activity. Knockdown of NKILA increased CTL infiltration and suppressed breast cancer progression (159).

The differentiation of Th cells can be regulated by lncRNAs. MALAT1 interacted with miR-155 as ceRNA and down-regulated the expression of miR-155 in CD4+ T cells. The ratio of Th1/Th 2 was adjusted through this pathway. Up-regulation of MALAT1 produced more Th2-type cytokines while inhibiting the release of Th1-type cytokines (160). Th17 cells are a newly discovered CD4+T cell subgroup associated with anti-tumor activities. The Th17 cell subgroup can theoretically make up for the deficiency of the Th1/Th2 mediated immune response mechanism in the human body. Treg cells that mediate immune tolerance and Th17 cells that mediate inflammatory response are in a state of resistance in function and differentiation (161). The process of CD4+ T cell differentiation into Th17 cells can be affected by lncRNA NEAT1. Knockdown of NEAT1 inhibited Th17/CD4+ T cell differentiation by reducing the level of STAT3 protein (162).

In the tumor microenvironment, T cells can induce and differentiate into regulatory T cells (Tregs). Different from Th17 cells, Tregs have an immunosuppressive function. They can inhibit the anti-tumor immunity of immune effector cells such as CD4+ T cells, cytotoxic CD8+ T cells, NK cells, DC, and other immune cells through various mechanisms to promote tumorigenesis and tumor development. Tregs are a critical factor in tumor immune escape and play a vital role in tumor immune regulation (163). Long non-coding RNAs have been involved in regulating the differentiation and function of Tregs. Lnc-epidermal growth factor receptor (EGFR), specifically bound to EGFR, blocked its interaction with c-CBL and c-CBL ubiquitination, upregulated its downstream AP-1/NF-AT1 axis, and then triggered EGFR Expression to promote Treg differentiation and HCC progression (164). In another report, exosomes (TDEs) derived from breast tumor cells can induce CD73+γδ1 Treg cells through the SNHG16/miR-16-5p/SMAD5 regulatory axis. LncRNA SNHG16 sponged miR-16-5p, suppressed the target gene SMAD5, and enhanced the TGF-β1/SMAD5 pathway, thereby upregulating the expression of CD73 in Vδ1 T cells (165). LncRNA fetal-lethal non-coding developmental regulatory RNA (FENDRR) upregulated growth arrest and DNA-damage-inducible beta protein (GADD45B) through sponge miR-423-5p to inhibit Treg-mediated immune escape of HCC cells (166). Although removing Treg cells can enhance the anti-tumor immune response, it may also trigger autoimmunity at the same time. A vital issue of cancer immunotherapy for Treg is to specifically deplete Treg cells infiltrating into tumor tissues while suppressing auto-immunity without affecting the anti-tumor effect of immune cells. Also, the functional balance between Th17 cells and Tregs is critical and can be an essential part of regulating immunity and cancer (39, 167).

The activation of NKT cells was also related to lncRNAs. LINC00240 overexpression inhibited the cytotoxic activity of NKT cells by the miR-124-3p/STAT3/MICA axis and enhanced the growth, migration, and invasion of cervical cancer cells (168). In summary, T cells play a critical part in immune responses and cancer development. T cells have a big family, and their functions vary from one to another. LncRNAs participate in the development and biological process of T cells. Therefore, exploring the intercommunications between the T cells and lncRNAs facilitate the regulation of cancer immunity and investigates the potential therapies of cancer.

#### 5.2.2 LncRNAs and B Cells

In the past research, the roles of T cells in tumor immunity have been extensively studied, while the research on B cells is relatively rare. B lymphocytes are derived from pluripotent stem cells. Stimulated by the antigens, B cells can differentiate into plasma cells. The plasma cells infiltrate the tumor site and produce various cytokines and antibodies, which participate in anti-tumor immunity *via* ADCC, phagocytosis, and complement activation. In addition, B cells can also drive antigens to CD4+ and CD8+ T cells to facilitate antigen-specific immune response (169, 170). In recent years, the role of lncRNAs in B cells development and differentiation, as well as B cell-derived malignant tumors such as diffuse large B-cell lymphoma (DLBCL) and B lymphocytic leukemia, have been discussed (171). Some lncRNAs have shown potential as therapeutic targets (**Figure 3**).

LncRNAs are related to B cells development. Mitotic cell cycle-related genes of B cells such as KIF23, PLK4, and CENPE associated with the lncRNAs OIP5-AS, MME-AS1, and the bidirectional lncRNA CRNDE (172–174). Diffuse large B-cell lymphoma is a type of tumor composed of medium to large B lymphoid cells. LncRNAs were reported to regulate the immune evasion of the tumor. Small nucleolar RNA host gene 14 (SNHG14) acted as a ceRNA to sponge miR-5590-3p, then increased the expression of Zinc finger E-box binding homeobox 1 (ZEB1) and induced PD1/PD-L1 activities. The SNHG14/miR-5590-3p/ZEB1 pathway facilitated the immune evasion of DLBCL cells and the progression of DLBCL. This study indicated that targeting lncRNAs may be potential immunotherapy in DLBCL (175). There were also examples of lncRNAs and DLBCL related to MYC. MYC proto-oncogene was upstream of lncRNA functional intergenic repeating RNA element (FIRRE). The lncRNA FIRRE promoted cell proliferation and reduced cell apoptosis in DLBCL *via* the Wnt/β-catenin signaling pathway, promoting DLBCL development (176). The balance between the B-cell precursors’ proliferation and apoptosis contributed to B-cell precursor acute lymphoblastic leukemia (BCP-ALL). Although the specific mechanism has not been clarified, lncRNAs were reported to affect tumor development. Long non-coding RNA colorectal neoplasia differentially expressed (LncRNA CRNDE) increased the expression of cyclic AMP response element-binding protein (CREB) *via* sponging miR-345-5p. Then the BCP-ALL cell proliferation was promoted, and cell apoptosis was downregulated (177). In another study, the anti-tumor roles of lncRNAs were revealed. LncRNA GAS5 overexpression sponged miR222. B lymphocytic leukemia cells were arrested in the G1 phase of the cell cycle, the cell proliferation and invasion of B lymphocytic leukemia was inhabited, and cell apoptosis was promoted (178). These studies showed that lncRNA expression was related to B cells and B cell-derived tumors development. The function of lncRNAs deserve further studies and may become potential clinical therapies.

### 5.3 LncRNAs and Stromal Cells in TIME

#### 5.3.1 LncRNAs and Cancer Stem Cells

Cancer stem cells (CSCs) refer to cancer cells with the properties of stem cells, which have the ability to self-renewal and differentiate. As for the origin, it has not yet been elucidated. Some normal tissue stem cells or progenitor cells may undergo oncogenic transformation to produce CSCs (179). The current view believes that CSCs have the following characteristics: (a) exist in the primary tumor; (b) can maintain self-renewal through asymmetric cell division; (c) can continuously proliferate and differentiate. The epithelial-mesenchymal transition (EMT) of CSC allows cancer cells to spread and metastasize from the primary tumor; (d) resistance to therapy (180, 181). In addition, increasing researches have confirmed the associations between CSCs and immune cells in the tumor microenvironment, including TAMs, DCs, T cells, and MDSCs. For example, CSCs secrete cytokines and chemokines, including CCL2, CCL5, CSF1, GDF15, IL-13, TGFβ, and Wnt-induced signaling protein 1 (WISP1), to recruit and polarize TAMs (182–184). CSCs can also reduce the anti-tumor effects of DCs by restricting their transport, preventing their maturation, and inducing the differentiation of tolerance subtypes (184, 185). Moreover, CSCs can directly interact with T cells. CSC partly reduces AKT and ERK signal transduction by releasing related extracellular vesicles or free tenascin C (TNC) to escape from anti-tumor T cells but induce tumor-promoting regulatory Tregs (184, 186). In addition, the interaction between CSCs and MDSCs in TME was also reported. CSCs can secrete soluble factors and exosomes to promote the MDSCs infiltration, expansion, and activation (184, 187). LncRNAs are involved in regulating the self-renewal and epithelial-mesenchymal transition (EMT) of CSC.

LncRNAs can modulate the CSC self-renewal function. For example, lncRNA HAND2-AS1 has reported high expression in liver CSCs. Mechanistically, HAND2-AS1 recruited the INO80 chromatin-remodeling complex to the promoter of BMPR1A to induce the expression of BMP signaling. Thereby the self-renewal of CSCs and tumorigenesis were promoted. Knockdown of lncRNA HAND2-AS1 provided a potential target for HCC therapy (188). In another study, the role of lncTCF7 in promoting CSCs self-renewal has also been reported. Different from the above mechanism, lncTCF7 recruited the SWI/SNF complex to the promoter of TCF7 to induce Wnt signaling expression (189). Moreover, lncRNAs participate in the EMT of CSCs, which promotes tumor metastasis. Long non-coding RNA HOTTIP was associated with the EMT of pancreatic cancer stem cells (PCSCs), suggesting a potential therapeutic target. Mechanistically, *via* HOTTIP/WDR5/HOXA9/Wnt axis, HOTTIP affected stemness, including sphericity, tumorigenesis, stem factors (LIN28, NANOG, OCT4, and SOX2), and markers (ALDH1, CD44, and CD133) (190). In another study, lncRNA H19 and miR-675 participated in enriching the CSC pool, facilitating stemness properties of breast cancer cells and tumor migration (191). HCC-associated mesenchymal stem cells (HCC-MSC) have contributed to EMT, and lncRNAs have participated in these processes. LncRNA-MUF was an MSC-upregulated factor, and the overexpression lncRNA-MUF could accelerate EMT and malignant capacity. Mechanistically, lncRNA-MUF interacted with Annexin A2 (ANXA2) to induce Wnt/β-catenin signaling expression and EMT process. Moreover, lncRNA-MUF also acted as a ceRNA for miR-34a to upregulate Snail1 and promote EMT. Depleting lncRNA-MUF suppressed EMT, and this lncRNA-mediated process was a potential method for therapeutic targeting (192).

#### 5.3.2 LncRNAs and Cancer-Associated Fibroblasts

Cancer-associated fibroblasts (CAFs) are the core components of TME, playing an essential role in the occurrence and development of tumors. CAFs are mainly derived from fibroblasts or stellate cells in the pancreas and liver tissues (193). Stimulation including TGF-β family ligands, lysophosphatidic acid (LPA), fibroblast growth factor (FGF), platelet-derived growth factor (PDGF), IL-1, and IL-6 can activate fibroblasts to become CAFs (194). The role of CAF is mainly to promote tumors, but in some cases, also to inhibit tumors (195, 196). CAFs can regulate blood vessel formation, immune response, tumor progression, chemotherapy, and radiation therapy *via* remodeling of ECM and secreting growth factors. They can reshape the ECM structure through the application of cross-linking enzymes, proteases, and forces. This may lead to the tracking of collagen, which can accelerate the migration of cancer cells. Another situation of changing ECM is that CAFs can generate high pressure and interstitial pressure to prevent treatment delivery, thereby hindering the effect of cancer treatment and compressing blood vessels. Besides, CAFs can also resist treatment by secreting soluble mediators. VEGF and hepatocyte growth factor (HGF) can regulate angiogenesis (193, 197). Moreover, by cytokine production and surface molecules, CAF can regulate immunity, including macrophage recruitment, T cells immune response, and DCs anti-tumor immunity (198). LncRNAs can regulate CAF activation, tumor progression, and chemoresistance.

LncRNAs participated in the reprogramming process of normal fibroblasts (NFs) into CAFs. LncRNA Gm26809 was delivered by melanoma cell B16F0-derived exosomes into NIH/3T3 cells to reprogram fibroblast NIH/3T3 into tumor-promoting CAFs. The proliferation and migration of melanoma cells were promoted. Moreover, the knockdown of Gm26809 reduced pro-tumor effect (199). In another study, lnc-CAF facilitated squamous cell carcinoma progression by reprogramming NFs into CAFs through lnc-CAF/IL-33 pathway. In this process, lnc-CAF increased the expression of IL-33 and inhabited p62-dependent autophagy-lysosome degradation of IL-33. A high level of lnc-CAF was related to tumor development. While lnc-CAF knockdown downregulated Ki-67 and α-SMA+ CAF expression, limited the tumor growth (200).

The long noncoding RNAs have been regarded as nodal drivers of metastatic progression mediated by CAFs. The role of LINC00092 in ovarian cancer aggressiveness has been demonstrated. LINC00092 interacted with a glycolytic enzyme named 6-phosphofructo-2-kinase/fructose-2,6-biphosphatase 2 (PFKFB2) to modulate glycolysis levels and supportive function of CAFs, thereby facilitating ovarian cancer metastasis (201). In addition, the exosomal LINC00659 transferred from CAFs were revealed to bind miR-342-3p and then upregulate ANXA2 expression. The proliferation, EMT, and migration of colorectal cancer (CRC) cells have been promoted (202).

The crosstalk between CAFs and cancer cells was associated with chemoresistance and lncRNAs participated in some of the processes. CAFs delivered exosomal lncRNA H19 to act as a ceRNA sponging miR-141 and then activated the β-catenin pathway. MiR-141 had an inhibitory effect on the stemness of CRC cells. Through the lncRNA H19 pathway, the stemness and chemoresistance of CRC were facilitated (203). Also, in another study, lncRNA CCAL was demonstrated to contribute to tumor chemoresistance. Exosomes delivered lncRNA CCAL from CAFs to the cancer cells. Then it bound to mRNA stabilizing protein HuR (human antigen R) to increase β-catenin mRNA and protein levels. The CRC cell apoptosis was suppressed, and the oxaliplatin (Oxa) resistance was promoted (204). These findings indicated that lncRNAs were involved the biological activities of CAFs. Moreover, lncRNAs have the potential to be the target for chemoresistance.

#### 5.3.3 LncRNAs and Endothelial Cells

Vascular endothelial cells (EC) can complete the metabolic exchange of plasma and tissue fluid, synthesize various biologically active substances for tissue development, and act as antigen-presenting cells to participate in immune activities (205, 206). The newly formed blood vessels can supply nutrients for the growing primary tumor. Meanwhile, the tumor cells synthesize and release various substances to accelerate angiogenesis, facilitating tumor progression (207, 208). Vascular endothelial growth factor (VEGF) acts as a mitogen and pro-angiogenic factor, promoting endothelial cell proliferation, increasing vascular permeability, and promoting endothelial cells to express PA, PAI, interstitial collagenase, and thrombin activity. Tumor angiogenesis, development, and metastases can be facilitated (209–211). LncRNAs can regulate angiogenesis by directly regulating endothelial cells’ development, differentiation, proliferation, migration, autophagy, and apoptosis. Also, the VEGF can be regulated to manage angiogenesis.

Long noncoding RNA SENCR contributed to the differentiation from pluripotent cells to the endothelial. The proliferation, angiogenic capacity, and migration ability of human umbilical endothelial cells (HUVEC) were associated with the expression of SENCR (212). In another study, the regulatory role of lncRNA MALAT1 in endothelial function and vessel growth has been demonstrated. Inhibiting lncRNA MALAT1 facilitated endothelial cell proliferation, migration, and angiogenesis *via* the PI3K/Akt signaling pathway (213). In another study, lncRNA was revealed to protect endothelial function *via* DNA damage response (DDR). LncRNA maternally expressed gene 3 (Meg3) interacted with the RNA binding protein polypyrimidine tract binding protein 3 (PTBP3) and activated p53 signaling. This pathway played a vital role in the cell apoptosis and cell proliferation triggered by the DDR. Endothelial homeostasis can be regulated through this pathway, which could be targeted in future therapies (214). What is more, lncRNA TGFB2-OT1 (TGFB2 overlapping transcript 1) derived from the 3’UTR of TGFB2 can regulate autophagy of ECs. As a ceRNA, TGFB2-OT1 sponged MIR3960, MIR4488 and MIR4459, to affect the miRNA targets CERS1 (ceramide synthase 1), NAT8L (N-acetyltransferase 8-like [GCN5-related, putative]), and LARP1 (La ribonucleoprotein domain family, member 1). CERS1 and NAT8L were involved in autophagy *via* regulating mitochondrial function. TGFB2-OT1 upregulated the expression of LARP1, which increased the SQSTM1 (sequestosome 1) level, NFKB RELA and CASP1 functions, and the secretion of IL6, IL8, and IL1B in VECs (215). Moreover, VEGFA expression can be upregulated by lncRNA LINC00173.v1 in squamous cell carcinoma (SQC) tissues. Suppression LINC00173.v1 downregulated the vascular endothelial cells proliferation and the SQC tumorigenesis. Mechanistically, LINC00173.v1 upregulated the VEGFA level *via* interacting with miR-511-5p. Adopting the antisense oligonucleotide (ASO) method to inhabit LINC00173.v1 was a potential strategy to limit SQC progression and upregulated the therapeutic sensitivity of SQC (216).

#### 5.3.4 LncRNAs and Cytokines in TIME

Cytokines are small molecular proteins synthesized and secreted by immune cells (such as monocytes, macrophages, T cells, B cells, NK cells, etc.) and specific non-immune cells (endothelial cells, epidermal cells, fibroblasts, etc.). Cytokines can be divided into transforming growth factor-β family (TGF-β), interleukin (IL), interferon (IFN), chemokine family, etc. They can mediate the interaction between cells and have a variety of biological functions in regulating the tumor microenvironment (217, 218).

TGF-β is a crucial immunosuppressive cytokine. TGF-β can regulate the production and function of a variety of immune cells. It controls the innate immune system by inhibiting NK cells and regulating macrophages and neutrophils to form a negative immune input. It also promotes the expansion of Treg cells, inhibits the generation and function of antigen-presenting dendritic cells and effector T cells, and directly regulates adaptive immunity (219, 220). The TGF-β signaling pathway and lncRNA network play a vital role in EMT cancer development (221). In one study, TGF-β activated the Smad pathway to participate in EMT. LncRNA EMT-associated lncRNA induced by TGFβ1 (ELIT-1) increased the TGFβ/Smad3 signaling and TGFβ target genes expression, including Snail (a transcription factor critical for EMT), *via* developing a positive feedback loop. Eventually, lncRNA ELIT-1 facilitated EMT and cancer progression (222). In another study, TGF-β interacted with LncRNA-ATB to promote EMT and hepatocellular carcinoma (HCC) metastasis. LncRNA-ATB sponged miR-200 family to increase expression of ZEB1 and ZEB2. In addition, lncRNA-ATB supported organ colonization of tumor cells by autocrine induction of IL-11 and activating STAT3 signaling. These findings suggested that lncRNA-ATB had the potential to be the target for cancer therapy (223). Another study recognized that TGF-β induced lncRNA AC026904.1 and UCA1 were closely correlated to poor prognosis. AC026904.1 was an enhancer RNA in the nucleus, and UCA1 acted as ceRNA in the cytoplasm. These two lncRNAs cooperated to increase the level of Slug, playing critical roles in TGF-β-induced EMT (224). Moreover, accumulating evidence suggested that TGF-β-induced EMT is NF-κB-dependent. Repression of NF-κB signaling downregulated TGF-β-induced EMT. LncRNA NKILA was upregulated by TGF-β and participated in the negative feedback loop of the NF-κB pathway. Overexpression of NKILA suppressed EMT and tumor metastasis, which may serve as a therapeutic target (225).

IL-6 is a pleiotropic cytokine expressed and distributed in a variety of cells. It regulates inflammation and cellular immune response and plays a role in tumor metabolism by activating various carcinogenic pathways (217). IL-6 is overexpressed in a variety of cancer cells, and cancer cells rely extensively on IL-6 signal transduction. IL-6 activates signal transducers and activators of transduction-3 (STAT3) to affect tumor-infiltrating immune cells, stimulate downstream target genes to protect tumor cells from apoptosis, facilitate tumor cell proliferation, upregulate tumor angiogenesis and drug resistance (226). For example, the oncogenic roles of IL-6/STAT3 signaling and lncRNAs have been demonstrated. Patients were exhibiting unsatisfying responses to sorafenib therapy in renal cell carcinoma (RCC) patients. LncRNA-SRLR (sorafenib resistance-associated lncRNA in RCC) was found upregulated in sorafenib-resistant RCCs. Mechanistically, lncRNA-SRLR interacted with NF-κB and enhanced IL-6 transcription, activating STAT3 and promoting sorafenib tolerance (227). However, there were anti-tumor lncRNAs negatively regulating IL-6/STAT3 signaling. Long noncoding RNA on chromosome 8p12 (termed TSLNC8) was frequently deleted in HCC. TSLNC8 was able to competitively bind with transketolase and STAT3, regulate the STAT3-Tyr705 and STAT3-Ser727 phosphorylation expression, repressing the IL-6-STAT3 signaling pathway and suppressing HCC progression and metastasis (228). Lnc-DILC also inhibited IL-6/STAT3 signaling. Mechanistically, lnc-DILC mediates the crosstalk between TNF-α/NF-κB signaling and autocrine IL-6/STAT3 cascade and connects hepatic inflammation with LCSC expansion, suggesting that lnc-DILC could be a potential prognostic biomarker and possible therapeutic target against LCSCs (229).

Interferons (IFNs) have an excellent inhibitory effect on the growth of a variety of tumors. The anti-tumor mechanism of interferon-alpha is to exert its cellular activity by binding to specific membrane receptors. Once the IFN is attached to the cell membrane, it conducts signal transduction through IFNAR1, and IFNAR2, triggering a series of intracellular processes, such as activating the JAK/STAT signaling pathway, to inhibit cancer cell proliferation, and regulate the function of the immune system (230). LncRNA IRF1-AS (Interferon Regulatory Factor 1 Antisense RNA) was an IFN-inducible nuclear lncRNA reported to repressed esophageal squamous cell carcinoma (ESCC) progression by promoting IFN response through a positive regulatory loop with IRF1 (Interferon Regulatory Factor 1). Mechanistically, IFNs upregulated IRF1-AS *via* the JAK-STAT pathway. IRF1-AS activates IRF1 transcription through interacting with ILF3 (Interleukin Enhancer Binding Factor 3) and DHX9 (DExH-Box Helicase 9) (231). Interferon-γ (IFN-γ) is essential for the innate immune response. Mechanistically, Sros1 blocked the binding of Stat1 mRNA to the RBP CAPRIN1, stabilizing the Stat1 mRNA and promoting IFN-γ-mediated activation of innate immune responses (232). Mineo et al. found that tumor IFN signal upregulated PD-L1 level to suppress T cell-mediated immune surveillance. Silencing lncRNA INCR1 reduced the function of PD-L1, JAK2, and IFNγ-stimulated genes. In addition, knocking out INCR1 made tumor cells susceptible to the killing effect of T cells, thereby making improvements in CAR-T (154).

CCL18 is mainly produced by innate immune cells, including dendritic cells, monocytes, and macrophages. CCL18 attracts naive T cells, regulatory T cells, Th2 cells, immunosuppressive and immature dendritic cells, and effector B cells. The expression of CCL18 was positively associated with HOTAIR and promoted tumor progression. Mechanistically, HOTAIR acted as a ceRNA for miR-130a-5p to derepress ZEB1, promoting EMT in esophageal squamous cell carcinoma (ESCC). CCL18 facilitated cancer development by upregulating HOTAIR expression, providing a potential new therapeutic target for cancer diagnosis and treatment (233).

## 6 Potential Applications for Cancer Diagnosis and Treatment

**Figure 4:**
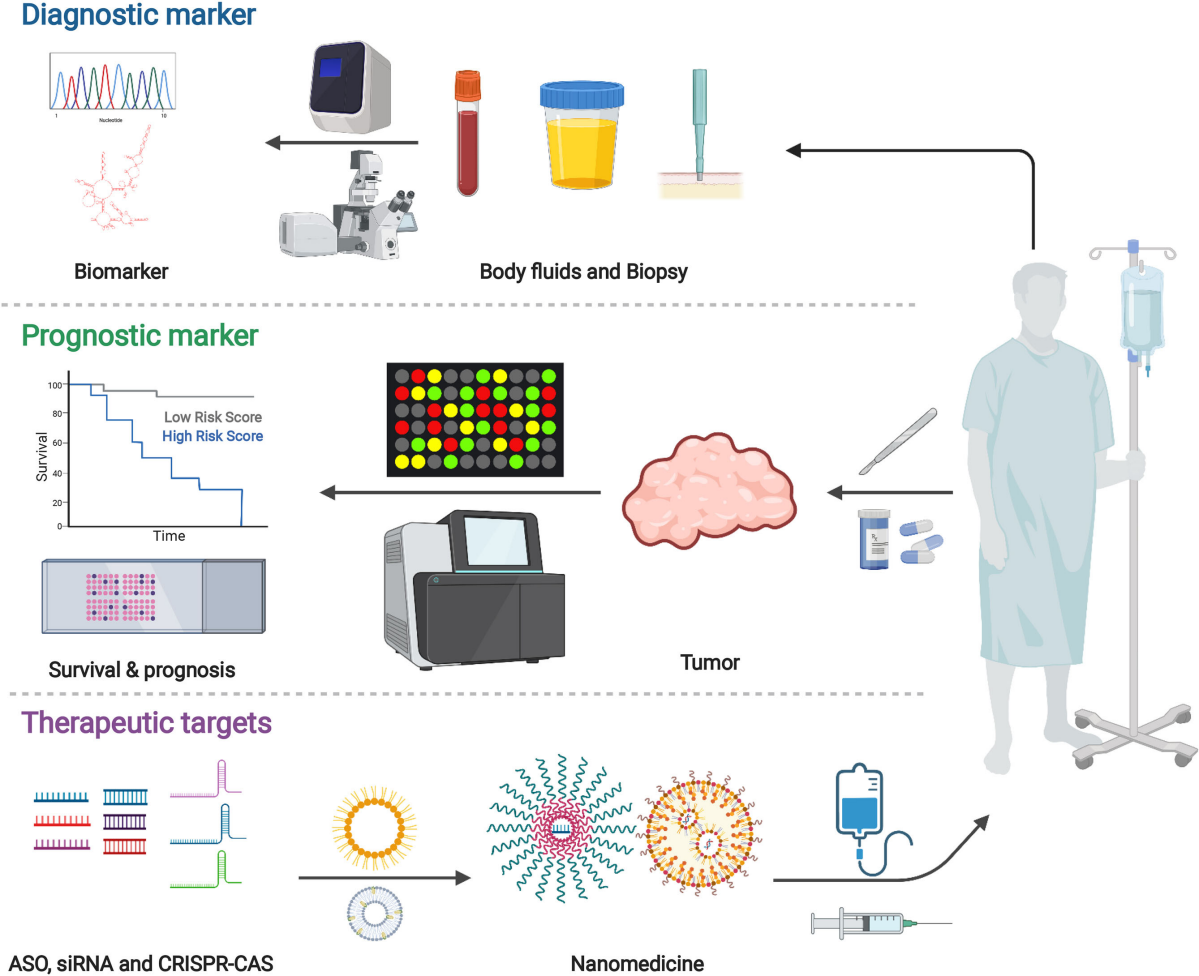
Potential applications for cancer diagnosis and treatment. Various findings demonstrated the importance and value of long non-coding RNAs. Studies highlighted the potential of lncRNA in biomarkers for cancer diagnosis and targets for cancer therapy. Created with BioRender.com.

LncRNAs have the characteristics of tissue-specific expression, and the differentially expressed lncRNAs can be used as cancer biomarkers. Moreover, lncRNAs remain relatively stable in circulating body fluids, which makes non-invasive detection possible. The detection of lncRNAs is also expected to bring more information than DNA detection. In addition, from a pharmaceutical perspective, long non-coding RNAs are also attractive targets because they can directly regulate disease-related gene expression. All in all, lncRNAs are closely related to tumorigenesis, tumor development, and metastasis. It is promising as a potential biomarker for tumor diagnosis and novel target for tumor treatment (**Figure 4**) (234, 235).

Biomarker includes various objective indications or medical signs which can be measured accurately and reproducibly. A prognostic biomarker influences the clinical outcome (236). Recent studies have shown that the abnormal expression of MALAT1 in tumor tissues and body fluids can be used as a biomarker for tumor diagnosis and prognosis. MALAT1 regulates various molecular signaling pathways, such as MAPK/ERK, PI3K/AKT, WNT/β-catenin, and NF-kB, to participate in immune response, angiogenesis and tumor progression (237). SNHG15 serves as an oncogenic regulator in cancer progression. The expression of SNHG15 was significantly related to the poor prognosis. SNHG15 functions as a ceRNA sponging miRNA in cancer, meditating oncogenic factors, thus ruling malignant phenotype and EMT. Studies have shown that SNHG15 participated in various signaling pathways essential for cancer, including EMT, WNT/β-catenin, NF-kb, and YAP-Hippo signaling pathways. SNHG15 may be a prospective and effective biomarker for cancer diagnosis (238).

LncRNAs-related immunotherapy has many advantages. Firstly, lncRNAs can regulate a series of downstream target genes by participating in various cell signaling pathways to regulate cancer treatment. In addition, the multiple regulatory sites of lncRNAs can interact with other molecules, which helps to develop new structure-based anticancer drugs (30). More importantly, as discussed before, lncRNAs played a crucial role in cancer immunology. They influenced many biological processes, including neutrophil recruitment, macrophage polarization, NK cells cytotoxicity, T cells functions, etc. The potential therapeutic applications of lncRNAs have attracted more and more attention.

Although various regulatory functions of lncRNAs have been revealed, one primary concern is how we can effectively and safely target these lncRNAs. Recently, studies have discussed the new technologies in lncRNA biology. According to the information from human genome sequencing and the base-pairing rules of Watson and Crick, it is possible to design therapeutic oligonucleotides (239–243). Arun et al. used antisense oligonucleotides (ASO) to systematically knock down Malat1, which slowed down tumor development in the mouse cancer model (244). RNA interference (RNAi) can silence lncRNA. Pichler et al. found that lncRNA FLANC induced angiogenesis through the STAT3/VEGFA pathway. Using nanoparticles that carry small interfering RNA to target FLANC can reduce angiogenesis. This may be a potential new type of cancer treatment (245). In addition, CRISPR/Cas9, as a newly discovered genome editing tool, Cas9 nuclease can delete the lncRNA gene or introduce RNA unstable elements into its locus (246). Zhu et al. introduced a method based on CRISPR/Cas9 utilizing paired-gRNAs (pgRNAs) to produce large-fragment deletions, which allowed the identification of functional lncRNAs in tumor cells (247). In another study, a CRISPR/Cas9 technology system named CERTIS (CRISPR-mediated Endogenous lncRNA Tracking and Immunoprecipitation System) was developed to visualize and separate endogenous lncRNA by precisely inserting an MS2 tag into the distal end of the lncRNA locus. By this method, lncRNA NEAT1 was effectively labeled and monitored for its endogenous expression variation (248).

In addition, recent advances revealed that regulation of the immune system and combined immunotherapies might be effective strategies (249, 250). For example, Li et al. identified a cancer immunogenic lncRNA named lncRNA inducing MHC-I, and immunogenicity of tumor (LIMIT) was involved in the MHC-I expression and T cells responses *via* LIMIT-GBP-HSF1 axis. LIMIT cis-activated the guanylate-binding protein (GBP) gene cluster, and GBPs disrupted the association between HSP90 and heat shock factor-1 (HSF1), thereby resulting in HSF1 activation and transcription of MHC-I (107). Immunotherapies such as Immune Checkpoint Blocking (ICB) and Chimeric Antigen Receptor T Cell Therapy (CAR-T) have been gradually applied to tumor treatment (30, 251). In recent years, immune checkpoint-targeting drugs, such as CTLA-4, PD-1, and PD-L1 have been utilized for cancer immunotherapy (44). In one study, H3K27 acetylation activated two immune checkpoint molecules, PD-L1 and galectin-9. LncMX1–215 worked as a tumor suppressor molecule in head and neck squamous cell carcinoma (HNSCC), showing its negative function in immunosuppression. Mechanistically, lncMX1–215 was bound to GCN5, a known H3K27 acetylase, to interference with the interaction with H3K27 acetylation (252). However, the application of (CAR)-T cells is limited due to poor tumor invasion, progression of failure, and insufficient antigen.

Another concern lies in the efficient delivery of lncRNAs. Although lncRNAs are attractive therapeutic targets, there are still some limitations. For example, the molecules can be rapidly degraded and cleared from circulation. Besides, the large size makes it difficult to pass through the cell membrane (253). Nowadays, various materials and approaches, including biomaterials, controlled release systems, and nanoparticles (254, 255). They also show the potential to deliver lncRNAs and target cells. For example, implantable biomaterials construct a structure to attract and reprogram DCs for immunotherapy. Ali et al. revealed that polymers had the potential to be structured for releasing cytokine, presenting cancer antigens, recruiting immune cells, and promoting immune responses (256, 257). There are also injectable biomaterials such as hydrogels, gelatin, and mesoporous silica micro rods. These materials are highly deformable and self-assembled without surgical implantation, creating a local immunogenic environment to recruit and activate immune cells. Ji et al. explored a hydrogel patch harboring LSD1 inhibitor and chemotherapy agent, which enhanced tumor immunogenicity and increased T-cell infiltration *via* epigenetic activation of innate immunity (258). This study revealed the broad applicability of epigenetic remodeling hydrogel patches, which also inspired the lncRNA-related strategies. Hori et al. developed a kind of alginate with rapid self-gelling property *in vivo*. This material was fabricated by the mixture of calcium-loaded alginate microspheres with soluble alginate solution and DCs. When the constructed material was injected into mice, the activated DCs in the alginate facilitated the recruit and immune responses of the T cells (259, 260). Nanoparticles have been adopted for the delivery of lncRNAs and targeting desired cells (261). Amita M Vaidya et al. developed RGD-PEG-ECO/siDANCR nanoparticles to facilitate delivery of siDANCR and then silence lncRNA DANCR, which was a therapeutic target for triple-negative breast cancer (TNBC). The results showed prolonged DANCR silencing and suppressing TNBC proliferation in mice with no overt side-effects (262). In another study, due to the biocompatibility and biodegradability of poly (lactic acid/glycolic) (PLGA), PLGA-based nanoparticles were applied to develop a drug delivery and controlled release system. LINC00958 was associated with lipogenesis that exacerbated HCC. Based on PLGA, a nanoplatform was fabricated to deliver si-LINC00958 for HCC suppression (263). Besides, there was a study about protein-mimicking nanoparticles, which was reported to modulate a cellular homeostasis without displaying a general toxicity (264), which may act as potential nanomedicines to combination lncRNA-based therapy in cancer treatment. Nucleic acids are not allowed to transfer biological membranes directly. However, the polymorphic lipid phase can temporarily compromise the permeability barrier and allow nucleic acids to enter the cell. Extracellular vesicles as therapeutic vehicles have stoked lots of interest (265, 266). *Via* EVs, lncRNAs were delivered between immune and tumor cells to play a regulatory role in cancer development. A previous study demonstrated that TAMs enhanced breast cancer cells’ aerobic glycolysis and apoptotic resistance by the EV delivery of HIF-1α-stabilizing long noncoding RNA (HISLA). Mechanistically, HISLA interrupted the reaction between PHD2 and HIF-1α, thereby suppressing the hydroxylation and degradation of HIF-1α (267).

## 7 Conclusions

The interactions between the immune system and tumor microenvironment are complex. LncRNAs have been demonstrated to participate in various cellular metabolic, immune cell activations, and immune responses. Moreover, they play a crucial role in regulating tumorigenesis, tumor progression, angiogenesis, and immunosuppression. Therefore, understanding the regulatory role of lncRNAs in the TIME can broaden the field of antitumor immunity. Targeting lncRNAs could be a promising clinical approach in cancer therapy. Herein, we critically reviewed various characters and regulatory roles of lncRNAs. Potential strategies include increasing infiltration of antitumor immune cells, enhancing the work efficiency of antigen-presenting cells, improving the toxicity of effector cells, and reprogramming immunosuppressive cells.

There are perspectives on the awaiting challenges and future directions of immunotherapies based on lncRNAs. So far, the proportion of patients who respond well to immunotherapy is still deficient. Only a few measures have been able to enter human clinical research. Therefore, understanding the molecular and cellular drivers of immune escape is significant. Besides, optimizing long-term survival with multi-agent cancer immunotherapy can be promising. The combination of immunotherapy and lncRNAs have the potential to promote tumor treatment. Moreover, it is expected to enhance the therapeutic effect of immunotherapy and reduce side effects.

## Author Contributions

YL designed this study. YG and YX drafted the manuscript. YL, YG, and YX revised the manuscript. All authors read and approved the final manuscript.

## Funding

This work was supported by the National Natural Science Foundation of China (No. 31771102, 82003262), China Postdoctoral Science Foundation (No. 2019TQ0221, 2019M663517), and Post-Doctor Research Project, West China Hospital, Sichuan University (No. 2019HXBH059).

## Conflict of Interest

The authors declare that the research was conducted in the absence of any commercial or financial relationships that could be construed as a potential conflict of interest.

## Publisher’s Note

All claims expressed in this article are solely those of the authors and do not necessarily represent those of their affiliated organizations, or those of the publisher, the editors and the reviewers. Any product that may be evaluated in this article, or claim that may be made by its manufacturer, is not guaranteed or endorsed by the publisher.
